# Metagenomic insights into the composition and function of the gut microbiota of mice infected with *Toxoplasma gondii*

**DOI:** 10.3389/fimmu.2023.1156397

**Published:** 2023-04-06

**Authors:** Jin-Xin Meng, Xin-Yu Wei, Huanping Guo, Yu Chen, Wei Wang, Hong-Li Geng, Xing Yang, Jiang Jiang, Xiao-Xuan Zhang

**Affiliations:** ^1^College of Veterinary Medicine, Qingdao Agricultural University, Qingdao, Shandong, China; ^2^College of Life Science, Changchun Sci-Tech University, Changchun, China; ^3^College of Animal Science and Veterinary Medicine, Heilongjiang Bayi Agricultural University, Daqing, Heilongjiang, China; ^4^State Key Laboratory for Animal Disease Control and Prevention, Harbin Veterinary Research Institute, Chinese Academy of Agricultural Sciences, Harbin, Heilongjiang, China; ^5^Department of Medical Microbiology and Immunology, School of Basic Medicine, Dali University, Dali, Yunnan, China

**Keywords:** *Toxoplasma gondii*, toxoplasmosis, gut microbiota, metagenomic sequencing, intestinal inflammation

## Abstract

**Introduction:**

Despite Toxoplasma gondii infection leading to dysbiosis and enteritis, the function of gut microbiota in toxoplasmosis has not been explored.

**Methods:**

Here, shotgun metagenomics was employed to characterize the composition and function of mouse microbial community during acute and chronic T. gondii infection, respectively.

**Results:**

The results revealed that the diversity of gut bacteria was decreased immediately after T. gondii infection, and was increased with the duration of infection. In addition, T. gondii infection led to gut microbiota dysbiosis both in acute and chronic infection periods. Therein, several signatures, including depression of Firmicutes to Bacteroidetes ratio and infection-enriched Proteobacteria, were observed in the chronic period, which may contribute to aggravated gut inflammation and disease severity. Functional analysis showed that a large amount of Kyoto Encyclopedia of Genes and Genomes (KEGG) pathways and carbohydrate-active enzymes (CAZy) family displayed distinct variation in abundance between infected and healthy mice. The lipopolysaccharide biosynthesis related pathways were activated in the chronic infection period, which might lead to immune system imbalance and involve in intestinal inflammation. Moreover, microbial and functional spectrums were more disordered in chronic than acute infection periods, thus implying gut microbiota was more likely to participate in disease process in the chronically infected mice, even exacerbated immunologic derangement and disease progression.

**Discussion:**

Our data indicate that the gut microbiota plays a potentially important role in protecting mice from T. gondii infection, and contributes to better understand the association between gut microbiota and toxoplasmosis.

## Introduction

*Toxoplasma gondii* is a type of parasite that survives in cells and is capable of infecting a vast majority of endotherms, including humans (1). The primary means of *T. gondii* contact is direct or indirect consumption of food and water containing *T. gondii* oocysts (2, 3). The initial manifestation of *T. gondii* infection is usually asymptomatic; however, in some cases, the infection may cause cervical lymphadenopathy or ocular disease (4). In particular, pregnant women infected with *T. gondii* may face severe harm to their fetus (5). Toxoplasmosis is one of the major global public health issues. Approximately 8%–22% of individuals in the United States and a similar ratio in the United Kingdom are estimated to be infected with *T. gondii*. Meanwhile, in Central America, South America, and continental Europe, the infection rates are believed to range from 30% to 90% (6, 7). In China, among 49,784 Chinese blood donors, the overall incidence of *T. gondii* infection is 6.26% as determined by immunoglobulin G (IgG) seroprevalence (8). Upon the momentous impact of toxoplasmosis on the health of humans and animals, it is urgent to develop effective treatment measures.

The actively proliferating tachyzoites of *T. gondii*, which give rise to acute infection, are usually effectively controlled by the body’s immune system (9, 10). Current studies suggest that there are complex interplays among *T. gondii*, the mucosal immune system, and the gut microbiota during parasitic infection (11, 12). The gut microbiota is considered as a significant ally of human cells. The engagement of the gut microbiota in interactions with human cells and the alteration of the gut microbiota balance have been implicated in disrupting human health (13–15). The maladjustment of intestinal microecology may cause many diseases, including those affecting the cardiovascular and nervous systems, and directly correlates to gastrointestinal disease (16). The alteration of the gut microbiota is strongly associated with the inflammatory responses and malfunction of the intestines during parasitic infection (17). Many parasites, such as *Blastocystis* sp. and *Cryptosporidium parvum*, can change the intestinal microbial community structure, which is able to influence the onset and progression of disease (17, 18). Research regarding the complex interplays among the immune system, gut bacteriome, and protozoa emphasizes the safeguarding role of the gut microbiota against protozoan infections (17, 19). In-depth understanding of this protective mechanism would help prevent and treat intestinal parasitic infections through changing the compositions of the intestinal flora.

Recently, some studies showed the diversity and composition of the gut microbiota in rodents infected with *T. gondii* by 16S amplicon sequencing (20–22). However, the functional features of the gut microbiota in mice with toxoplasmosis remain unclear. To investigate the potential role of the gut microbiota in mice against *T. gondii* infection, we explored the dynamic changes of intestinal bacteria during *T. gondii* infection periods (acute and chronic periods) using metagenomic sequencing. Insights into aspects of structure and function of the microbial community may help researchers and clinicians prevent and treat parasitic infections by manipulating microbiota components.

## Materials and methods

### Experimental design and sample collection

Eight- to 10-week-old female C57BL/6 mice with an average weight of 19 g were purchased from SPF (Beijing) Biotechnology Co., Ltd. All mice were raised under a 12-h light/dark cycle with an independent ventilation system and were given food and water by *ad libitum* access. The Prugniaud strain of *T. gondii* (Type II) used for constructing infectious models was obtained from the State Key Laboratory of Veterinary Etiological Biology. After 1 week of acclimatization, the mice were randomly divided into four groups (n = 6 per group): acute infection (AI) group, chronic infection (CI) group (CI), and two control groups for acute (AC) and chronic (CC) phases. The mice were orally infected with 20 *T. gondii* oocysts in the two infection groups and were perfused with 0.5 mL sterile phosphate-buffered solution in the control groups. The mice were sacrificed on day 11 (acute period; AI, AC) and day 33 (chronic period; CI, CC) after infection, and at least 2 g of cecal contents were aseptically collected and stored at -80°C for DNA extraction. All animal experiments were approved by the Qingdao Agriculture University Ethics Committee.

### DNA extraction and metagenomic sequencing

Genomic DNA was extracted from cecal contents using the cetyltrimethylammonium ammonium bromide method. The purity and concentration of DNA were determined by 1% agarose gel electrophoresis. The sequencing library was generated using NEB Next^®^ Ultra™ DNA Library Prep Kit for Illumina (NEB, USA) following the manufacturer’s recommendations, and index codes were added to each sample. Briefly, the genomic DNA sample was fragmented to a size of 350 bp by sonication for Illumina sequencing. Shotgun metagenomic sequencing was carried out on the Illumina NovaSeq 6000 platform with a 150-bp paired-end read length.

### Metagenome assembly and binning

Metagenomic sequence reads were filtered to exclude adapter and low-quality sequences using FASTP (v0.23.0) (23) with default parameters. All clean sequence reads were assembled by MEGAHIT (v1.2.9) (24). Reads were mapped to contigs with a length greater than 2,000 bp using the BWA MEM program (v0.7.17-r1188) (25). A total of 2,369 bins were generated by using MetaBAT2 (V.2.12.1) (26) with options “-m 2000 -s 200000 –save Cls –seed 2021”. dRep (v2.5.4) (27) program was used to remove redundancy at an average nucleotide identity (ANI) >99%. The remaining bins were evaluated using lineage_wf workflow of CheckM (v.1.1.2) (28). The taxonomic classification of the metagenome-assembled genomes (MAGs) was achieved using GTDB-Tk (v1.7.0) (29) and GTDB database (release 202).

### Species-level clustering, phylogenetic analysis, and abundance profiling

All MAGs were further clustered using dRep program with the principle that an ANI >95% resulted in 156 representative species-level genome bins (SGBs). Prokka (v1.14.5) (30) and PhyloPhlAn (v.1.0) (31) were applied to annotate the genomes and build a phylogenetic tree, respectively. The phylogenetic tree was annotated and visualized using iTOL (32). To profile the abundance of the SGBs, we aligned the clean reads onto the genome catalog by using bowtie2 (v2.2.5) (33) with default parameters, and the mapped read count of the SGBs was normalized to transcripts per kilobase million (TPM). The profiles of phyla and family were calculated by aggregating the relative abundance of the SGBs based on taxonomic ranks.

### Construction of the gene catalog and functional annotation

The contigs of the assembled sequences longer than 500 bp were used to predict open reading frames (ORFs) using Prodigal (v2.6.3) with procedure “meta” (34). Gene ORFs with a length <100 bp were removed and dereplicated by clustering at 90% aligned region with 90% nucleotide identity using MMseqs2 (v11.e1a1c) (35) with the parameters easy-cluster –min-seq-id 0.90 -c 0.9 –cluster-mode 2, yielding a nonredundant gene catalog containing 5,234,851 ORFs. We counted the reads that aligned to the gene catalog, and read count was normalized to TPM. All genes in our catalog were annotated to the Kyoto Encyclopedia of Genes and Genomes (KEGG) orthology (KO) (36) and CAZy family (37) using DIAMOND (v0.9.32) (38), default parameter except that –min-score 60 –query-cover 50.

### Statistical analyses

Statistical analyses were performed in an R 4.1.1 environment. The Shannon index was assessed based on the relative abundance profiles at the species level. The principal coordinate analysis (PCoA) based on the Bray–Curtis distance and the permutational multivariate analysis of variance (PERMANOVA) were used to evaluate the beta diversity (vegan package, v2.5-7). The Wilcoxon rank-sum test was performed to evaluate the significant difference in the diversity index and relative abundance of taxa, KOs, KEGG pathways, and CAZy family between diseased and healthy mice. Rarefaction analysis was performed to assess the gene richness in each group. Heatmap was generated in R with the *ComplexHeatmap* (v2.8.0) packages (39). All other demonstrations were generated using the ggplot2 (v3.3.5) package.

## Results

### Reconstruction of the 241 draft microbial genomes from cecal contents of mice infected with *T. gondii*

**Figure 1:**
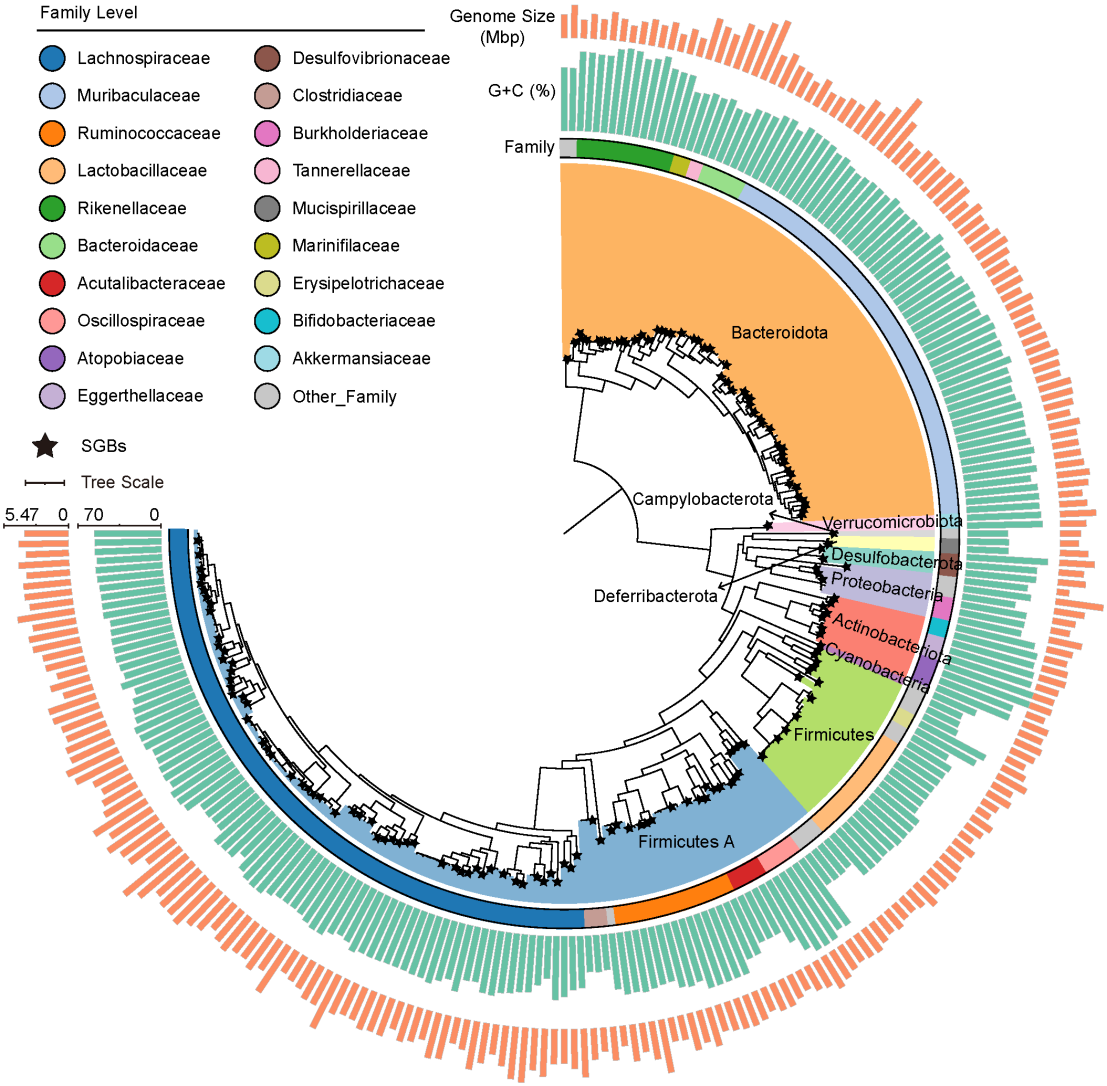
Phylogenetic tree of 241 metagenome-assembly genomes (MAGs) from the cecal contents of mice, with color filling on branches representing phylum-level classification. The stars mark the caudal end of the branches, indicating species-level genome bins (SGBs). The three rings from inside to outside represent family-level classification, GC content, and genome size of each MAG, respectively.

After DNA extraction, whole-genome sequencing, host filtering, and quality control steps, more than 164.79 Gb clean data were produced from 24 fresh cecal content samples (6.87 ± 0.49 Gb per sample, ST1). These metagenomic data were used for assembly and binning. A total of 2,369 raw bins were recovered. In order to obtain strict and representative MAGs, 1,584 dereplicated genomes (ANI ≥99%) were left, and then thresholds of ≥70% genome completeness, ≤5% contamination, and quality score (defined as completeness –5× contamination) <55 were used, resulting in 241 MAGs (ST2). To derive the view of the microbial community at the species level, 241 MAGs were organized into SGBs at an ANI threshold of 95%, resulting in a total of 156 SGBs for subsequent analyses. Based on taxonomic annotations from the Genome Taxonomy Database (GTDB), all genomes were annotated to family level, 234 genomes to genus, and 144 genomes to species. The bacterial genome catalog represented 10 phyla, 31 families, and 78 genera (**Figure 1**, ST2).

### Alteration of gut microbial diversity in mice experiencing *T. gondii* infection

**Figure 2:**
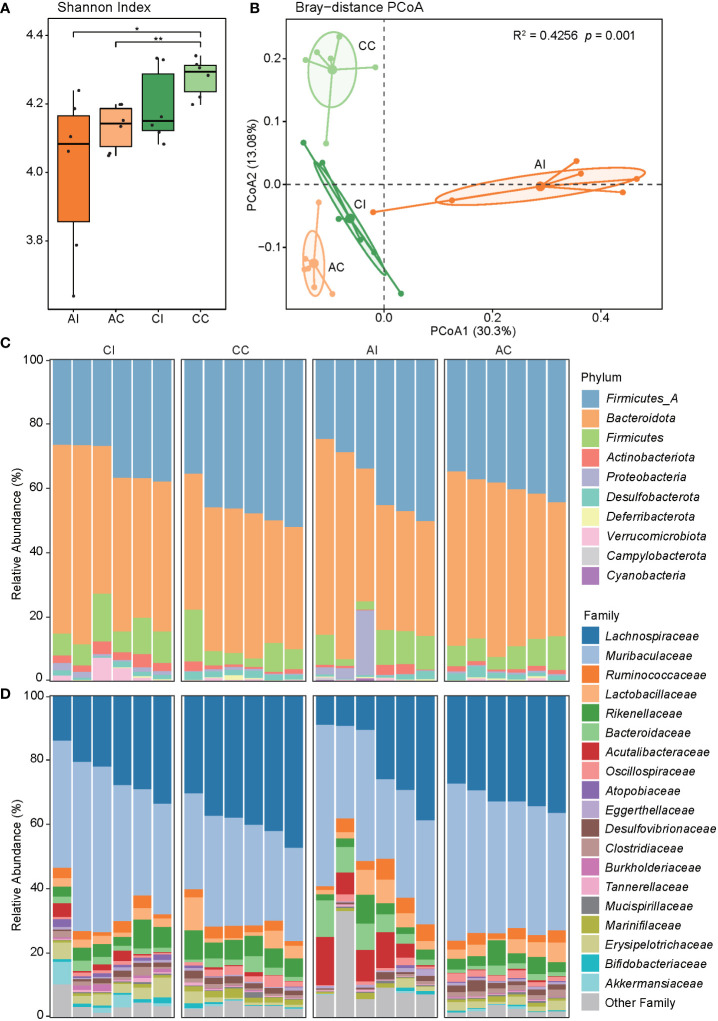
The diversity and composition of the microbial community. **(A)** The Shannon index of the mouse gut microbiota in each group, and asterisks indicate a statistical significance: * indicates *p* < 0.05; ** indicates *p* < 0.01. **(B)** PCoA shows the beta diversity of the microbial community. Community differences are verified by PERMANOVA. **(C, D)** The bar chart shows the taxonomic composition at the phylum and family levels.

We aligned the clean reads onto the genome catalog to quantify and profile the relative abundance of phyla, families, and species in order to investigate the diversity of the gut bacteriome in the mice. The Shannon index was calculated to measure the alpha diversity of the gut microbiota. In general, the diversity of gut bacteria was decreased after *T. gondii* infection and was increased with the duration of infection (CC vs. AC: *p* < 0.01, **Figure 2A**). PCoA showed that the PC 1 and PC 2 represented 30.30% and 13.08% of variance, respectively, and revealed a substantial difference in microbial community (PERMANOVA, R^2^ = 0.43, *p* < 0.001; AI vs. AC: R^2^ = 0.40, *p* < 0.002; CI vs. CC: R^2^ = 0.28, *p* < 0.002; **Figure 2B**). In addition, the cluster of the AI group was at the right in the panel and relatively farther from other clusters. These results indicated that the obvious clinical symptoms of acute *T. gondii* infection may be in part attributed to the changes of the gut microbiome in mice, and the microbial community had a stronger variation at acute than late stages of infection.

### Dysbiosis of the gut microbiome in diseased mice

**Figure 3:**
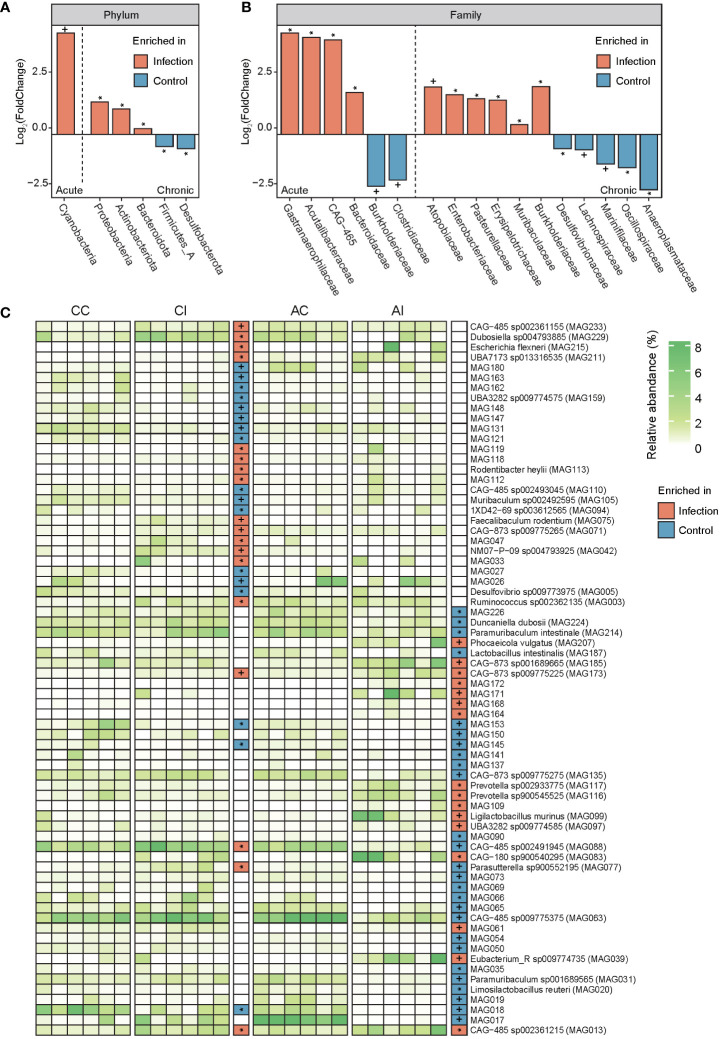
Comparison of the gut microbiota between infected and control groups at the phylum, family, and species levels. **(A, B)** Bar plots show the value of log_2_ (FoldChange) of each feature (phyla and family) that has a significant difference. Orange displays taxa enriched in the infected group, and light blue indicates taxa enriched in the control group. The left part of each panel (left of the dashed line) represents the acute period of infection, and the right part of each panel (right of the dashed line) represents the chronic period of infection. Signatures indicate a statistical significance: * indicates *p* < 0.05; + indicates *p* < 0.01. **(C)** The heatmap displays the relative abundance of each MAG in each sample. The white to green transition denotes low to high relative abundance. In grids of intermediate and the rightmost parts, orange indicates features enriched in the infected group and light blue indicates features enriched in the control group. Signatures indicate a statistical significance: * indicates *p* < 0.05; + indicates *p* < 0.01.

Bacterial profiles at different classification levels were used to uncover the microbial structure in guts of diseased and healthy mice. The results showed that *Bacteroidetes* and *Firmicutes* were dominant phyla in both groups, and the relative abundance of phyla *Actinobacteriota*, *Proteobacteria*, and *Desulfobacterota* was slightly more than 1% (**Figure 2C**, ST3). *Lachnospiraceae* and *Muribaculaceae* were dominant families, approximately accounting for two-thirds of the total abundance (**Figure 2D**, ST3). We further discovered that the relative abundance of five phyla, 11 families, and 35 species was significantly different between the CI and CC groups, while one phylum, six families, and 42 species were significantly different between the AI and AC groups (*p* < 0.05, **Figures 3A–C**, ST4). In the chronic period, the ratio of *Firmicutes* to *Bacteroidetes* was decreased in the infected animals, but the abundance of *Actinobacteriota* and *Proteobacteria* was significantly increased (*p* < 0.05). Nevertheless, no similar trends were seen in the acute period. At the family level, *Acutalibacteraceae*, *Bacteroidaceae*, *CAG-465* (belonged to class *Clostridia*), and *Gastranaerophilaceae* were significantly enriched, whereas *Burkholderiaceae* and *Clostridiaceae* were depleted in the infection group compared to the control group in the acute phase. In addition, *Atopobiaceae*, *Burkholderiaceae*, *Enterobacteriaceae*, *Erysipelotrichaceae*, *Muribaculaceae*, and *Pasteurellaceae* were enriched in CI mice, and *Anaeroplasmataceae*, *Lachnospiraceae*, *Muribaculaceae*, *Marinifilaceae*, and *Oscillospiraceae* were enriched in the CC group. We observed that 16 and 18 species were enriched in the AI and CI groups, respectively. For example, *Parasutterella sp900552195* (MAG077), assigned to Gammaproteobacteria class, was visibly enriched in the infection group in the chronic phase but was depleted in the acute stage. Therefore, our findings indicated that *T. gondii* infection induced alterations of the gut microbial community.

### Functional alteration of the gut microbiome in diseased mice

**Figure 4:**
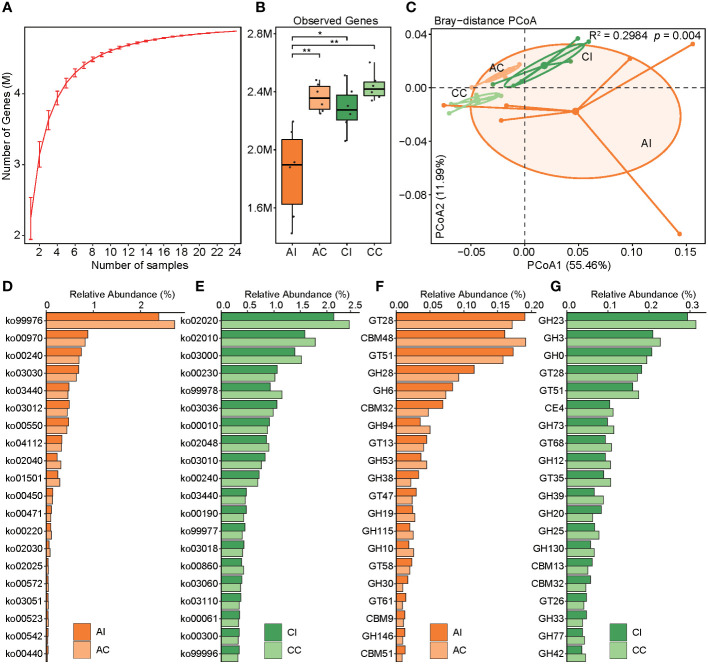
Construction of the gene catalog and comparison of the microbial function. **(A)** Rarefaction curves for the gene number in the gene catalog after 100 random samplings. **(B)** Comparison of the microbial gene count in the four groups, and asterisks indicate a statistical significance: * indicates *p* < 0.05; ** indicates *p* < 0.01. **(C)** PCoA based on the Bray–Curtis distance of the KO profile shows the beta diversity of microbial function. Microbial functional differences were verified by PERMANOVA. **(D, E)** Bar plots show the relative abundance of the top 20 remarkably different KEGG pathways (*p* < 0.05). Deep orange indicates the infected group in the acute period, and light orange indicates control. Deep green indicates the infected group in the chronic period, and light green indicates control. **(F, G)** Bar plots show the relative abundance of the top 20 remarkably different CAZy family (*p* < 0.05). Deep orange indicates the infected group in the acute period, and light orange indicates control. Deep green indicates the infected group in the chronic period, and light green indicates control.

To characterize the microbial function against *T. gondii* infection in mice, a gene catalog was constructed and gene abundance was profiled. The rarefaction curve tended to attain the saturation plateau, suggesting that the sequencing data were great enough to detect majority of the genes (**Figure 4A**). We noted that the number of genes was decreased after infection at both acute and chronic stages (AI vs. AC, *p* < 0.01; **Figure 4B**). PCoA based on the KO profile revealed apparent differences in microbial functions between pre- and post-infection (PERMANOVA, R^2^ = 0.30, *p* < 0.004; AI vs. AC: R^2^ = 0.21, *p* < 0.0.089; CI vs. CC: R^2^ = 0.36, *p* < 0.003; **Figure 4C**). In total, 1,305 and 1,873 out of 8,006 KOs were differentially abundant between infected individuals and controls in the acute and chronic periods, respectively (*p* < 0.05, ST5). The corresponding 43 and 86 KEGG pathways showed significant differences in the acute and chronic stages, respectively (**Figures 4D, E**; ST). Overall, in the acute infection phase, we found 19 pathways that had higher abundance in diseased mice, including lipid metabolism (three pathways), metabolism of other amino acids (three pathways), glycan biosynthesis and metabolism (two pathways), etc., whereas 24 pathways were significantly enriched in healthy cohorts, mainly including pathways related to metabolism (eight pathways), environmental information processing (four pathways), and cellular processes (four pathways). At the chronic infection stage, the abundance of 49 pathways was significantly higher in diseased mice than that in healthy ones, such as metabolism of cofactors and vitamins (five pathways), signal transduction (four pathways), amino acid metabolism (three pathways), and biosynthesis of other secondary metabolites (three pathways). Nonetheless, 37 pathways were remarkably enriched in healthy individuals. Intriguingly, homologous recombination (ko03440) and pyrimidine metabolism (ko00240) pathways of the gut microbiota in mice were significantly enriched in the phase of acute *T. gondii* infection, and this effect could last until 33 days. DNA repair- and synthesis-related pathways were apparently activated, perhaps reflecting a strategy utilized by the gut microbiota to contend with environment stress after infected intervention. However, the cell motility-related pathway was depleted in diseased mice in the whole infection period (ko02030, ko02040). In terms of CAZy families, 38 and 23 enzymes were significantly enriched or depleted in the AI group, respectively (*p* < 0.05; **Figures 4F, G**; ST6). In the chronic infection phase, 44 enzymes were significantly enriched in the controls (*p* < 0.05), whereas 54 enzymes had the opposite trend. It had become clear that changes of the microbial function were larger in chronic compared to acute infection periods.

## Discussion

The cardinal symptom of *T. gondii* infection is inflammatory response, and the progression of disease is related to the gut microbiota (40). Although a growing body of research has characterized the diversity and structure of the gut microbiota in hosts infected with *T. gondii*, little is known about gut microbial functions against *T. gondii* infection in mice. Metagenomic sequencing can map out a landscape of microbial functions and understand the relationship between microbial genes and functions, exhibiting more advantages compared to 16S amplicon sequencing. Therefore, we characterized the gut bacterial community using the mouse *T. gondii* infection model in acute and chronic periods and further analyzed the microbial function before and after infections. In this study, a *T. gondii* infection-related genome catalog of mouse microbiome representing 241 MAGs and 156 SGBs was recovered, which provided us the species-level perspective to understand the relationship between the mouse microbiome and *T. gondii* infection. In addition, a nonredundant gene catalog (5,234,851 genes) was constructed to comprehensively understand the association between *T. gondii* infection and microbial function.

The diversity of gut bacteria was decreased after *T. gondii* infection and was increased over time (**Figure 2A**). Despite the temporal sampling strategy being different from this study, a previous study demonstrated that the bacterial diversity was decreased in the *T. gondii*-infected intestine after 5 days compared to the controls and was increased after 5 months (40). Another study considered that the diversity of the gut microbiota was increased after 13 days in infected mice, and the diversity was less than that of the controls after 21 days (20). These inconsistencies may be due to the surviving environment, infective dose, different parasite genotypes, and sampling time. It can confirm that the diversity of the gut microbiota is visibly altered after *T. gondii* infection.

Moreover, PCoA revealed significant variation in the gut microbial communities of mice in both acute and chronic infection periods (**Figure 2B**). In line with previous studies, the structure and composition of the gut microbiota showed a dramatic variation after *T. gondii* infection (20, 22, 40). Previous studies have shown that during *T. gondii* infection, *Firmicutes*, *Proteobacteria*, and *Bacteroidetes* were the primarily affected phyla. However, the *Actinobacteriota* was found to be susceptible after *T. gondii* infection in this study. There are a few studies investigating the relation of *Actinobacteriota* and parasitic infection. In the chronic infection period, a reduced ratio of *Firmicutes* and *Bacteroidetes* was observed in the gut of infected mice compared to that of healthy mice (**Figure 2C**). A decreasing ratio of *Firmicutes* and *Bacteroidetes* is associated with a variety of enteric diseases and intestinal inflammation, such as irritable bowel syndrome and inflammatory bowel disease (IBD) (41–43). Nevertheless, a similar phenomenon was not observed in the acute period. We observed a higher abundance of opportunistic pathogens in the mouse gut after infection, such as *Parasutterella*, *Rodentibacter*, and *Escherichia* of phylum *Proteobacteria* in the catalog (44–47). In fact, some evidence proved that the relative abundance of *Proteobacteria* was associated with an ecological imbalance in the gut microbial community of enteritis hosts and could be used as a diagnostic marker for the instability of the gut microbiota (48). Additionally, evidence shows a positive relationship between the severity of intestinal inflammation and the abundance of *Proteobacteria* (49–51). In particular, Wang et al. (52) considered that the bacterial dysbiosis and enrichment of *Proteobacteria* and *Enterobacteriaceae* in the distal small intestine of mice infected orally by *T. gondii* were mediated by CD4^+^ T cells, and when depleting CD4^+^ T cells, the pathology and microbial community were ameliorated. It was a valid scientific evidence that supported the hypothesis of complex interplays among *T*. gondii, microbes, and the host immune system. As anticipated, *Enterobacteriaceae* was enriched in both infected periods and may be attributed to this interaction effect. Despite the gut microbiota having substantial variations in both acute and chronic periods, it is of concern that few detected taxa had similar expression patterns (enrichment or depletion in the same direction) whether at family or species level. A possible explanation was that gut microbiota played distinct roles during the infection process. More reasonable longitudinal experiments and manipulation of the microbiota in the *T. gondii*-infected mice should be performed in the future to address this limitation.

The gene repertoire showed a decreased trend of the observed gene number in infected individuals compared to that of healthy cohorts and an increasing trend over time. Some studies have reported that bacterial community compositions varied significantly as the change in overall gene richness, and a loss of gene richness was associated with IBD (53, 54). Although no case of low microbial gene richness in *T. gondii* infection has been reported, the trends of gene richness and diversity of microbiota were remarkably consistent and might be associated with gut inflammation and dysbiosis. To the best of our knowledge, gut microbial functions in mice after infection have not been explored yet. We found that microbial functions were remarkably altered in infected mice whether in acute or chronic infected periods as compared with those in control mice, and the functions in diseased mice varied even more at the chronic infected stage. A large number of KOs and KEGG pathways were differentially abundant between infected cohort and healthy control. In the chronic infected period, an enrichment of genes involved in lipopolysaccharide (LPS) biosynthesis in infected mice was observed. LPS, a pro-inflammatory microbial product, can stimulate cytokine cascades and caspase activation, which are mediated by Toll-like receptor (TLR)-4. It not only causes local intestinal inflammation but also, along with cytokines, makes its way across the damaged barrier into the circulation, thus causing systemic inflammation (55–57). Intestinal inflammation about clinical presentation of post-infection was possibly attributed to a larger number of LPS-related genes bound with inflammasome sensors.

Thus, one possible explanation was that enteritis caused by *T*. gondii infection may be attributed to the accumulation of LPS produced by the gut microbiota. And we need to find more specific experimental evidence to confirm or reject this hypothesis. If so, the key target of microbes and microbial genes will be identified to prevent and control intestinal inflammation, even to remedy. In addition, bacterial toxins (ko02042) and pathogenic *Escherichia coli* infection (ko05130) pathways were significantly enriched in chronically infected mice, which may favor microbial infection (58). According to the aforementioned findings, we observed that families *Enterobacteriaceae* and *Escherichia flexneri* were significantly enriched in chronically diseased mice (**Figures 3B, C**; ST4). A higher abundance of pathways for pathogenic *E. coli* infection was consistent with findings in the aspect of species composition, which may contribute to gut inflammation and functional dysbiosis after *T. gondii* infection (59). In another interpretation, the interferon-gamma (IFN-γ)/Signal transducer and activator of transcription 1 (STAT1)/inducible nitric oxide synthase (iNOS) axis was activated against *T*. *gondii* during infection; meanwhile, host-derived nitrate also gave assistance to the expansion of *Enterobacteriaceae via* nitrate respiration (52). Unfortunately, no sufficient relevant evidence was found for our study of function to verify this result, although the reduction of the nitrate-related gene had somewhat different contents between per- and post-infection due to a lack of experimental facts. Moreover, a great deal of metabolic pathways was changed between infected and healthy groups. Consequently, modulating dietary or colonizing beneficial bacteria for controlling amino acid and energy intakes may provide a strategy to affect host metabolism, thereby preventing or treating toxoplasmosis. In terms of the CAZy family, for example, GT19 (lipid-A-disaccharide synthase) and GT30 (3-deoxy-D-manno-octulosonic-acid transferase) that were enriched in chronically infected mice were involved in LPS biosynthesis, which appears to be the cause of intestinal inflammation (60, 61).

In conclusion, we characterized taxonomic and functional signatures of the microbial community that associated with *T. gondii* acute infection. Our findings indicated that microbial and functional spectrums were more disordered in the chronic period compared with that in the acute period. In addition, we speculated several indications that might in part contribute to exacerbate intestinal inflammation and disease severity, including depression of the *Firmicutes*-to-*Bacteroidetes* ratio, infection-enriched *Proteobacteria*, and activation of LPS biosynthesis-related pathways. Therefore, we propose a hypothesis that both modulation of the composition of beneficial and harmful bacteria and inhibition of LPS biosynthesis might ameliorate toxoplasmosis through inhibiting inflammation. Future experiments will aim to verify this hypothesis. Taken together, our findings broaden our previous knowledge and provide new insights into temporal variations of microbial functions, which may facilitate to prevent or treat parasitic infections.

## Data availability statement

The original contributions presented in the study are publicly available. This data can be found here: https://db.cngb.org/search/project/CNP0003889/.

## Ethics statement

The animal experiments were approved by Qingdao Agriculture University Ethics Committee.

## Author contributions

X-XZ designed the study. J-XM, X-YW, YC, WW, H-LG, XY, JJ, and HG performed the research. J-XM, X-YW, and HG carried out the experiment, analyzed data and wrote the paper. All authors contributed to the writing and revisions. All authors contributed to the article and approved the submitted version.

## References

[1] Kyan H Takara T Taira K Obi T. Toxoplasma gondii antibody prevalence and isolation in free-ranging cats in Okinawa, Japan. J Vet Med Sci (2021) 83:1303–5. doi: 10.1292/jvms.21-0038 PMC8437723 34219071

[2] Zhang X Lou Z Huang S Zhou D Jia W Su C. Genetic characterization of toxoplasma gondii from qinghai vole, plateau pika and Tibetan ground-tit on the qinghai-Tibet plateau, China. Parasites Vectors (2013) 6:291. doi: 10.1186/1756-3305-6-291 24192458 PMC3852027

[3] Zhao X Ewald SE. The molecular biology and immune control of chronic toxoplasma gondii infection. J Clin Invest (2020) 130:3370–80. doi: 10.1172/JCI136226 PMC7324197 32609097

[4] Remington Jack S Thulliez P Montoya Jose G. Recent developments for diagnosis of toxoplasmosis. J Clin Microbiol (2004) 42:941–5. doi: 10.1128/JCM.42.3.941-945.2004 PMC356902 15004036

[5] Sanchez SG Besteiro S. The pathogenicity and virulence of toxoplasma gondii. Virulence (2021) 12:3095–114. doi: 10.1080/21505594.2021.2012346 PMC8667916 34895084

[6] Aguirre AA Longcore T Barbieri M Dabritz H Hill D Klein PN. The one health approach to toxoplasmosis: Epidemiology, control, and prevention strategies. EcoHealth (2019) 16:378–90. doi: 10.1007/s10393-019-01405-7 PMC6682582 30945159

[7] Bai M-J Zou Y Elsheikha HM Ma J-G Zheng W-B Zhao Q. Toxoplasma gondii infection in farmed wild boars (Sus scrofa) in three cities of northeast China. Foodborne Pathog Dis (2017) 14:379–85. doi: 10.1089/fpd.2016.2260 28387529

[8] Wang T Han Y Pan Z Wang H Yuan M Lin H. Seroprevalence of toxoplasma gondii infection in blood donors in mainland China: a systematic review and meta-analysis. Parasite (2018) 25:36. doi: 10.1051/parasite/2018037 30040610 PMC6057739

[9] Egan CE Cohen SB Denkers EY. Insights into inflammatory bowel disease using toxoplasma gondii as an infectious trigger. Immunol Cell Biol (2012) 90:668–75. doi: 10.1038/icb.2011.93 PMC4094106 22064707

[10] Cong W Huang S-Y Zhou D-H Zhang X-X Zhang N-Z Zhao Q. Prevalence and genetic characterization of toxoplasma gondii in house sparrows (Passer domesticus) in lanzhou, China. Korean J Parasitol (2013) 51:363–7. doi: 10.3347/kjp.2013.51.3.363 PMC3712113 23864750

[11] Cervantes-Barragan L Cortez VS Wang Q McDonald KG Chai JN Di Luccia B. CRTAM protects against intestinal dysbiosis during pathogenic parasitic infection by enabling Th17 maturation. Front Immunol (2019) 10:1423. doi: 10.3389/fimmu.2019.01423 31312200 PMC6614434

[12] Snyder LM Denkers EY. From initiators to effectors: Roadmap through the intestine during encounter of toxoplasma gondii with the mucosal immune system. Front Cell Infect Microbiol (2021) 10:614701. doi: 10.3389/fcimb.2020.614701 33505924 PMC7829212

[13] Cani PD. Human gut microbiome: hopes, threats and promises. Gut (2018) 67:1716. doi: 10.1136/gutjnl-2018-316723 29934437 PMC6109275

[14] Guida F Turco F Iannotta M De Gregorio D Palumbo I Sarnelli G. Antibiotic-induced microbiota perturbation causes gut endocannabinoidome changes, hippocampal neuroglial reorganization and depression in mice. Brain Behav Immun (2018) 67:230–45. doi: 10.1016/j.bbi.2017.09.001 28890155

[15] Shreiner AB Kao JY Young VB. The gut microbiome in health and in disease. Curr Opin Gastroenterol (2015) 31:69–75. doi: 10.1097/MOG.0000000000000139 25394236 PMC4290017

[16] Gu B-H Kim M Yun C-H. Regulation of gastrointestinal immunity by metabolites. Nutrients (2021) 13(1):167. doi: 10.3390/nu13010167 33430497 PMC7826526

[17] Partida-Rodríguez O Serrano-Vázquez A ME N-R Moran P Rojas L Portillo T. Human intestinal microbiota: Interaction between parasites and the host immune response. Arch Med Res (2017) 48:690–700. doi: 10.1016/j.arcmed.2017.11.015 29290328

[18] Mishra PK Palma M Bleich D Loke P Gause WC. Systemic impact of intestinal helminth infections. Mucosal Immunol (2014) 7:753–62. doi: 10.1038/mi.2014.23 24736234

[19] Kapczuk P Kosik-Bogacka D Kupnicka P Metryka E Simińska D Rogulska K. The influence of selected gastrointestinal parasites on apoptosis in intestinal epithelial cells. Biomolecules (2020) 10(5):674. doi: 10.3390/biom10050674 32349424 PMC7277436

[20] Shao DY Bai X Tong MW Zhang YY Liu XL Zhou YH. Changes to the gut microbiota in mice induced by infection with toxoplasma gondii. Acta Trop (2020) 203:105301. doi: 10.1016/j.actatropica.2019.105301 31843385

[21] Taggart PL Liddicoat C Tong WH Breed MF Weinstein P Wheeler D. Gut microbiota composition does not associate with toxoplasma infection in rats. Mol Ecol (2022) 31:3963–70. doi: 10.1111/mec.16552 PMC9546062 35621391

[22] Lv Q-B Ma H Wei J Qin Y-F Qiu H-Y Ni H-B. Changes of gut microbiota structure in rats infected with toxoplasma gondii. Front Cell Infect Microbiol (2022) 12:969832. doi: 10.3389/fcimb.2022.969832 35967867 PMC9366923

[23] Chen S Zhou Y Chen Y Gu J. Fastp: an ultra-fast all-in-one FASTQ preprocessor. Bioinformatics (2018) 34:i884–90. doi: 10.1093/bioinformatics/bty560 PMC6129281 30423086

[24] Li D Liu C-M Luo R Sadakane K Lam T-W. MEGAHIT: an ultra-fast single-node solution for large and complex metagenomics assembly *via* succinct de bruijn graph. Bioinformatics (2015) 31:1674–6. doi: 10.1093/bioinformatics/btv033 25609793

[25] Li H Durbin R. Fast and accurate short read alignment with burrows–wheeler transform. Bioinformatics (2009) 25:1754–60. doi: 10.1093/bioinformatics/btp324 PMC2705234 19451168

[26] Kang DD Li F Kirton E Thomas A Egan R An H. MetaBAT 2: an adaptive binning algorithm for robust and efficient genome reconstruction from metagenome assemblies. PeerJ (2019) 7:e7359. doi: 10.7717/peerj.7359 31388474 PMC6662567

[27] Olm MR Brown CT Brooks B Banfield JF. dRep: a tool for fast and accurate genomic comparisons that enables improved genome recovery from metagenomes through de-replication. ISME J (2017) 11:2864–8. doi: 10.1038/ismej.2017.126 PMC5702732 28742071

[28] Parks DH Imelfort M Skennerton CT Hugenholtz P Tyson GW. CheckM: assessing the quality of microbial genomes recovered from isolates, single cells, and metagenomes. Genome Res (2015) 25:1043–55. doi: 10.1101/gr.186072.114 PMC4484387 25977477

[29] Chaumeil P-A Mussig AJ Hugenholtz P Parks DH. GTDB-tk: a toolkit to classify genomes with the genome taxonomy database. Bioinformatics (2020) 36:1925–7. doi: 10.1093/bioinformatics/btz848 PMC7703759 31730192

[30] Seemann T. Prokka: rapid prokaryotic genome annotation. Bioinformatics (2014) 30:2068–9. doi: 10.1093/bioinformatics/btu153 24642063

[31] Segata N Börnigen D Morgan XC Huttenhower C. PhyloPhlAn is a new method for improved phylogenetic and taxonomic placement of microbes. Nat Commun (2013) 4:2304. doi: 10.1038/ncomms3304 23942190 PMC3760377

[32] Letunic I Bork P. Interactive tree of life (iTOL) v5: an online tool for phylogenetic tree display and annotation. Nucleic Acids Res (2021) 49:W293–6. doi: 10.1093/nar/gkab301 PMC8265157 33885785

[33] Langmead B Salzberg SL. Fast gapped-read alignment with bowtie 2. Nat Methods (2012) 9:357–9. doi: 10.1038/nmeth.1923 PMC3322381 22388286

[34] Hyatt D Chen G-L LoCascio PF Land ML Larimer FW Hauser LJ. Prodigal: prokaryotic gene recognition and translation initiation site identification. BMC Bioinf (2010) 11:119. doi: 10.1186/1471-2105-11-119 PMC2848648 20211023

[35] Steinegger M Söding J. MMseqs2 enables sensitive protein sequence searching for the analysis of massive data sets. Nat Biotechnol (2017) 35:1026–8. doi: 10.1038/nbt.3988 29035372

[36] Kanehisa M Goto S. KEGG: kyoto encyclopedia of genes and genomes. Nucleic Acids Res (2000) 28:27–30. doi: 10.1093/nar/28.1.27 10592173 PMC102409

[37] Drula E Garron M-L Dogan S Lombard V Henrissat B Terrapon N. The carbohydrate-active enzyme database: functions and literature. Nucleic Acids Res (2022) 50:D571–7. doi: 10.1093/nar/gkab1045 PMC8728194 34850161

[38] Buchfink B Xie C Huson DH. Fast and sensitive protein alignment using DIAMOND. Nat Methods (2015) 12:59–60. doi: 10.1038/nmeth.3176 25402007

[39] Gu Z. Complex heatmap visualization. iMeta (2022) 1:e43. doi: 10.1002/imt2.43 PMC10989952 38868715

[40] Prandovszky E Li Y Sabunciyan S Steinfeldt CB Avalos LN Gressitt KL. Toxoplasma gondii-induced long-term changes in the upper intestinal microflora during the chronic stage of infection. Scientifica (2018) 2018:2308619. doi: 10.1155/2018/2308619 30515345 PMC6236704

[41] Manichanh C Rigottier-Gois L Bonnaud E Gloux K Pelletier E Frangeul L. Reduced diversity of faecal microbiota in crohn’s disease revealed by a metagenomic approach. Gut (2006) 55:205. doi: 10.1136/gut.2005.073817 16188921 PMC1856500

[42] Collins SM. A role for the gut microbiota in IBS. Nat Rev Gastroenterol Hepatol (2014) 11:497–505. doi: 10.1038/nrgastro.2014.40 24751910

[43] Gophna U Sommerfeld K Gophna S Doolittle WF Veldhuyzen Van Zanten Sander JO. Differences between tissue-associated intestinal microfloras of patients with crohn's disease and ulcerative colitis. J Clin Microbiol (2006) 44:4136–41. doi: 10.1128/JCM.01004-06 PMC1698347 16988016

[44] Rizzatti G Lopetuso LR Gibiino G Binda C Gasbarrini A. Proteobacteria: A common factor in human diseases. BioMed Res Int (2017) 2017:9351507. doi: 10.1155/2017/9351507 29230419 PMC5688358

[45] Chen Y-J Wu H Wu S-D Lu N Wang Y-T Liu H-N. Parasutterella, in association with irritable bowel syndrome and intestinal chronic inflammation. J Gastroenterol Hepatol (2018) 33:1844–52. doi: 10.1111/jgh.14281 29744928

[46] Fingas F Volke D Hassert R Fornefett J Funk S Baums CG. Sensitive and immunogen-specific serological detection of rodentibacter pneumotropicus infections in mice. BMC Microbiol (2019) 19:43. doi: 10.1186/s12866-019-1417-7 30777007 PMC6380038

[47] Hoogland ICM Houbolt C van Westerloo DJ van Gool WA van de Beek D. Systemic inflammation and microglial activation: systematic review of animal experiments. J Neuroinflamm (2015) 12:114. doi: 10.1186/s12974-015-0332-6 PMC4470063 26048578

[48] Walker AW Sanderson JD Churcher C Parkes GC Hudspith BN Rayment N. High-throughput clone library analysis of the mucosa-associated microbiota reveals dysbiosis and differences between inflamed and non-inflamed regions of the intestine in inflammatory bowel disease. BMC Microbiol (2011) 11:7. doi: 10.1186/1471-2180-11-7 21219646 PMC3032643

[49] Maharshak N Packey CD Ellermann M Manick S Siddle JP Huh EY. Altered enteric microbiota ecology in interleukin 10-deficient mice during development and progression of intestinal inflammation. Gut Microbes (2013) 4:316–24. doi: 10.4161/gmic.25486 PMC3744516 23822920

[50] Peterson DA McNulty NP Guruge JL Gordon JI. IgA response to symbiotic bacteria as a mediator of gut homeostasis. Cell Host Microbe (2007) 2:328–39. doi: 10.1016/j.chom.2007.09.013 18005754

[51] Rehman A Lepage P Nolte A Hellmig S Schreiber S Ott SJ. Transcriptional activity of the dominant gut mucosal microbiota in chronic inflammatory bowel disease patients. J Med Microbiol (2010) 59:1114–22. doi: 10.1099/jmm.0.021170-0 20522625

[52] Wang S El-Fahmawi A Christian David A Fang Q Radaelli E Chen L. Infection-induced intestinal dysbiosis is mediated by macrophage activation and nitrate production. mBio (2019) 10:e00935–19. doi: 10.1128/mBio.00935-19 PMC6538788 31138751

[53] Gevers D Kugathasan S Denson Lee A Vázquez-Baeza Y Van Treuren W Ren B. The treatment-naive microbiome in new-onset crohn’s disease. Cell Host Microbe (2014) 15:382–92. doi: 10.1016/j.chom.2014.02.005 PMC4059512 24629344

[54] Weng YJ Gan HY Li X Huang Y Li ZC Deng HM. Correlation of diet, microbiota and metabolite networks in inflammatory bowel disease. J Dig Dis (2019) 20:447–59. doi: 10.1111/1751-2980.12795 31240835

[55] Man SM. Inflammasomes in the gastrointestinal tract: infection, cancer and gut microbiota homeostasis. Nat Rev Gastroenterol Hepatol (2018) 15:721–37. doi: 10.1038/s41575-018-0054-1 PMC7097092 30185915

[56] Candelli M Franza L Pignataro G Ojetti V Covino M Piccioni A. Interaction between lipopolysaccharide and gut microbiota in inflammatory bowel diseases. Int J Mol Sci (2021) 22:6242. doi: 10.3390/ijms22126242 34200555 PMC8226948

[57] Pasternak BA D'Mello S Jurickova II Han X Willson T Flick L. Lipopolysaccharide exposure is linked to activation of the acute phase response and growth failure in pediatric crohn's disease and murine colitis. Inflamm Bowel Dis (2010) 16:856–69. doi: 10.1002/ibd.21132 PMC3052288 19924809

[58] do Vale A Cabanes D Sousa S. Bacterial toxins as pathogen weapons against phagocytes. Front Microbiol (2016) 7:42. doi: 10.3389/fmicb.2016.00042 26870008 PMC4734073

[59] Zeng MY Inohara N Nuñez G. Mechanisms of inflammation-driven bacterial dysbiosis in the gut. Mucosal Immunol (2017) 10:18–26. doi: 10.1038/mi.2016.75 27554295 PMC5788567

[60] Bohl HO Shi K Lee JK Aihara H. Crystal structure of lipid a disaccharide synthase LpxB from escherichia coli. Nat Commun (2018) 9:377. doi: 10.1038/s41467-017-02712-9 29371662 PMC5785501

[61] Mamat U Schmidt H Munoz E Lindner B Fukase K Hanuszkiewicz A. WaaA of the hyperthermophilic bacterium aquifex aeolicus is a monofunctional 3-Deoxy-d-manno-oct-2-ulosonic acid transferase involved in lipopolysaccharide biosynthesis *. J Biol Chem (2009) 284:22248–62. doi: 10.1074/jbc.M109.033308 PMC2755949 19546212

